# Molecular evidence of *Anaplasma phagocytophilum* in olive baboons and vervet monkeys in Kenya

**DOI:** 10.1186/s12917-021-03095-2

**Published:** 2021-12-14

**Authors:** Sophie Jerusa Masika, Gerald Mwangi Muchemi, Tequiero Abuom Okumu, Samson Mutura, Dawn Zimmerman, Joseph Kamau

**Affiliations:** 1 Department of Public Health, Pharmacology and Toxicology, Faculty of Veterinary Medicine, University of Nairobi, Nairobi, Kenya; 2 Molecular biology laboratory, Institute of Primate Research, Nairobi Kenya, Nairobi, Kenya; 3 Global Health Program, Smithsonian Conservation Biology Institute, Washington, DC USA; 4 Department of Epidemiology of Microbial Disease, Yale School of Public Health, New Haven, CT USA

**Keywords:** Zoonosis, Olive baboons, Vervet monkeys, Kenya, *Anaplasma phagocytophilum*

## Abstract

**Background:**

Nonhuman primates (NHPs) play a significant role in zoonotic spill-overs, serving as either reservoirs, or amplifiers, of multiple neglected tropical diseases, including tick-borne infections. *Anaplasma phagocytophilum* are obligate intracellular bacteria of the family Anaplasmatacae, transmitted by Ixodid ticks and cause granulocytic anaplasmosis (formerly known as Human Granulocytic Ehrlichiosis (HGE)) in a wide range of wild and domestic mammals and humans too. The aim of this study was to determine whether *Anaplasma phagocytophilum* was circulating in olive baboons and vervet monkeys in Laikipia County, Kenya.

**Results:**

Some 146 blood samples collected from olive baboons and 18 from vervet monkeys from Mpala Research Center and Ol jogi Conservancy in Laikipia County were screened for the presence of *Anaplasma* species using conventional Polymerase Chain Reaction (PCR), and then *A. phagocytophilum* was confirmed by sequencing using conventional PCR targeting 16S rRNA. This study found an overall prevalence of 18.3% for *Anaplasma* species. DNA sequences confirmed *Anaplasma phagocytophilum* in olive baboons for the first time in Kenya.

**Conclusion:**

This study provides valuable information on the endemicity of *A. phagocytophilum* bacteria in olive baboons in Kenya. Future research is needed to establish the prevalence and public health implications of zoonotic *A. phagocytophilum* isolates and the role of nonhuman primates as reservoirs in the region.

## Background

There has been a rise in the frequency of emerging infectious diseases (EIDs), among which zoonotic tick-borne infections—especially rickettsial diseases such as anaplasmosis—are implicated [1]. What most of the recent pandemics have proven is that emerging infectious human diseases are mainly zoonosis of animal origin, particularly wildlife [2]. A complex series of interactions among wildlife, livestock and human populations and environmental factors contribute to their emergence [3].

Among wildlife species, non-human primates (NHPs) are often proprietors to many different microbial agents, some which have zoonotic potential. Primates are closely related to humans phylogenetically and ecologically [4], and they can indirectly transmit infectious agents to humans through intermediate hosts, arthropod vectors or directly through contact with or bites from the NHP, or through the consumption of NHP bush meat [5]. Certain factors such as forested tropical regions experiencing land-use changes and encroachment, as well as those with a high wildlife biodiversity, facilitate the spread of these diseases to livestock and man [6]. Others include adoption of new technology in farms, destruction of native forest habitats, climate change, global travel and human encroachment into new habitats [2].

This study focused on *Anaplasma phagocytophium,* a tick-borne pathogenic bacterium of zoonotic potential often harbored by wildlife, then passed into livestock and man [7]. *Anaplasma* belong to the family of *Anaplasmataceae*, order of Rickettsiales, class Alphaproteobacteria and genus *Anaplasma* [8]. This bacterium has a wide host range including domestic animals, wildlife and humans [9]. In the latter, the disease called human granulocytic anaplasmosis (HGA) (formerly known as human granulocytic ehrlichiosis [10] often presents with influenza-like symptoms which include fever, anorexia, diarrhea, leukopenia and thrombocytopenia [7]. Ixodid ticks are important in their maintenance as vectors [11]. The emergence of Anaplasmataceae as human pathogens has gained the attention of scientific community. Recent surveys have shown human infections of *Anaplasma* species in humans including *A. platys* in two women in Venezuela [12] and *A. phagocytophilum* exposure in dog owners in Morocco [13].

Recent reports on *A. phagocytophilum* infection in several animal species are available from France, Massachusetts, Brazil, Zambia, Ethiopia, and Kenya in both domestic and wild animals [14–19]. While *Anaplasma* in NHPs has been reported in some countries [17], its importance in NHPs in Kenya is not yet known which has therefore led us to investigate the occurrence of this bacteria in NHPs in Laikipia, Kenya.

The investigation focused on the detection of *A. phagocytophilum* DNA in blood samples from olive baboons (*Papio anubis*) and Vervet monkeys (*Chlorocebus pygerythrus*) in Laikipia County, Kenya. Laikipia is part of Kenya’s rangelands mainly inhabited by trans-human pastoralists and has a large wildlife population including non-human primate species found close to human settlements. The complex interaction among wildlife, livestock and human populations contributes to the emergence of infectious diseases [3] alongside factors that facilitate disease spread [2, 6].

The objectives of this present study were to (i) establish the presence of *A. phagocytophilum* in olive baboons and vervet monkeys and (ii) establish whether the bacteria is genetically diverse.

## Methods

### Study area, sample population and sample size

**Fig. 1:**
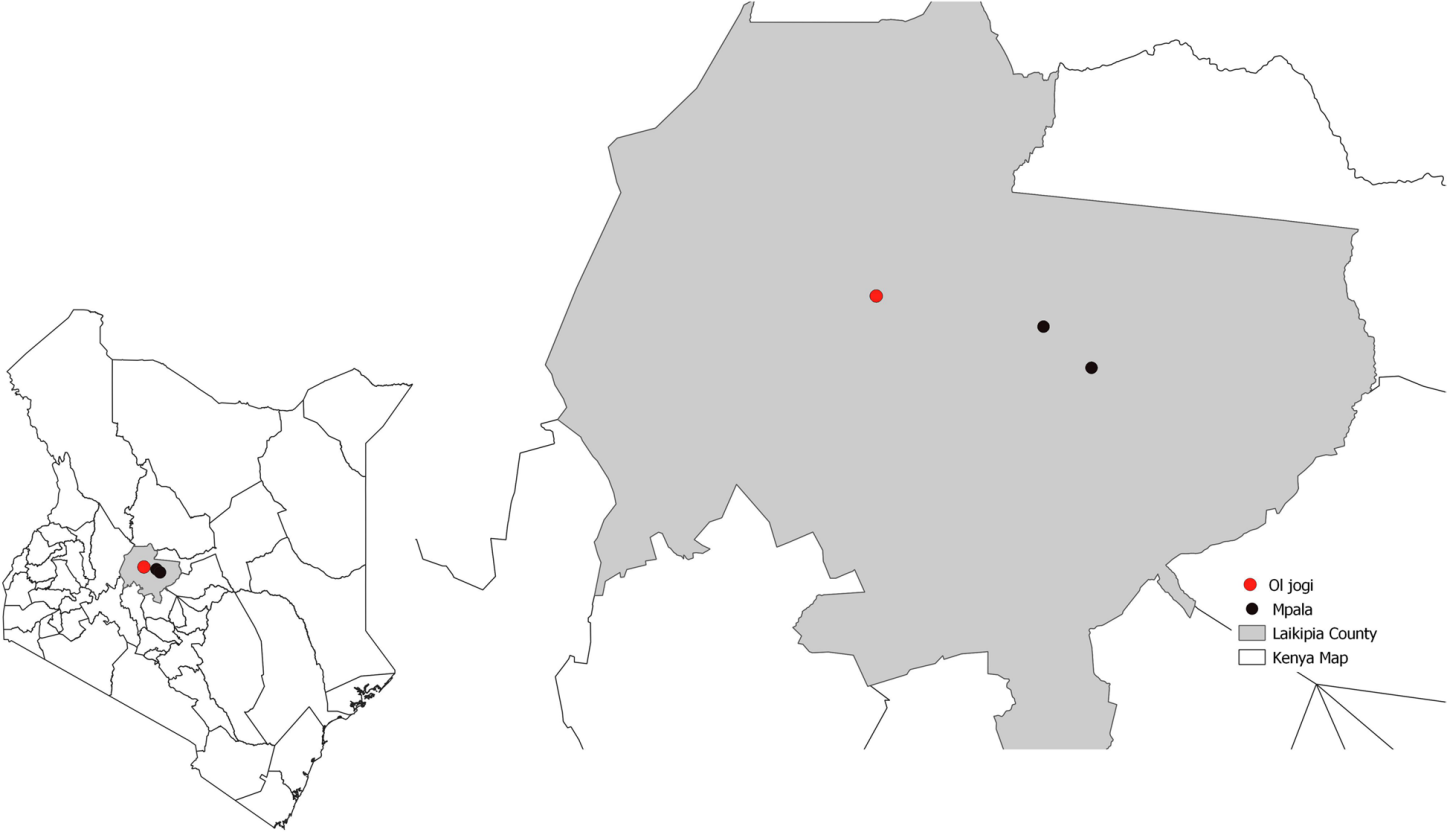
Map of the NHPs sampling sites in Laikipia County, Kenya

The study area was based on a larger project (USAID Predict II) whose aim was to collect targeted information to support the interventions to mitigate spread of zoonotic viruses with pandemic potential. The focus was on highest risk locations and interfaces, where animals and people share changing landscapes. The study area, Laikipia County (Fig. 1) located in the Rift Valley of Kenya (005′N 36040′E), was picked as one of the locations. There is a diverse range of wildlife in the area, including eight NHPs found in this area.

This study focused on two NHP species: olive baboons and vervet monkeys, since they are closely related to humans phylogenetically and are implicated in most of the listed emerging human pathogens [20]. The sampling sites Mpala research Centre and Ol jogi Conservancy (Fig. 1) were conveniently chosen since large populations of these species inhabit these sites. Some of the whole blood samples from wild olive baboons and vervet monkeys that had been collected by the larger project from Laikipia County were included in this study constituting the study’s sample size of 146 olive baboon and 18 vervet monkey. The animals were not owned.

### DNA extraction

Whole blood samples stored in TRIzol reagent at -80 °C were retrieved and allowed to thaw at room temperature. Extraction of genomic DNA was individually achieved from each whole blood samples using the DNeasy Blood & Tissue Kit (QIAgen Valencia, California, USA) following manufacturer’s instructions.

#### Targeted *PCR*

Of the extracted DNA, 2.0ul was used as template in the PCR reaction, targeting a 345 bp fragment of the *Anaplasma* 16S rRNA gene, using the EHR16SD/R primer set and previously defined conditions [21]. Amplicons were visualized on 1% agarose gels to validate amplicon size by comparison with a DNA molecular weight marker (1 Kb Plus DNA Ladder, Promega, Madison, USA).

### DNA sequencing and data analysis

Ten selected positive *Anaplasma* spp. PCR products obtained with primers EHR16SD/R were purified with Thermo Scientific GeneJET PCR Purification Kit #K0701, #K0702 Protocol according to the manufacturer’s instructions. Purified DNA fragments were sequenced using an ABI PRISM 377 Genetic Analyzer (Applied Biosystems, USA), using the original PCR primers (Table 1). Sequence assembly of forward and reverse chromatograms was done using DNA Sequence Assembler v.4 (2013), Heracle BioSoft [22]. The sequences were matched to those deposited in the GenBank database using the BLAST search (http://blast.ncbi.nlm.nih.gov/Blast.cgi). Multiple alignment of the sequences was done using BioEdit Sequence Alignment Editor (Hall, 1999). Construction of phylogenetic tree was done using Muscle 3.8 using the neighbor-joining method and visualization of the trees with FigTree v1.4.4 [23].

**Table 1:** Primers used for detection and/or characterization of *Anaplasma* species in the present study

Assay	Primer	Sequence 5′ to 3′	Target gene	Amplicon size (bp)	References
Conventional PCR	EHR16SD EHR16SR	GGTACCYACAGAAGAAGTCC TAGCACTCATCGTTTACAGC	16S rRNA	345	[3]

## Results

### Molecular survey of Anaplasma species

A total of 164 NHP blood samples—146 from olive baboons and 18 from vervet monkeys— were screened for the presence of *Anaplasma spp* through conventional PCR using primers targeting 345 bp of the 16S rRNA gene (Table 1). The overall prevalence for *Anaplasma* spp. was 18.3% (30/164) as estimated by EHR16SD/R PCR (Table 1), with 17.8 and 22.2% positivity rates in olive baboons and vervet monkeys respectively. Through sequencing, *A. phagocytophilum* was confirmed in three of ten gel positive samples, all from olive baboons from Mpala, Laikipia.

### Molecular characterization of *Anaplasma phagocytophilum*. 16S rRNA genotypes

**Fig. 2:**
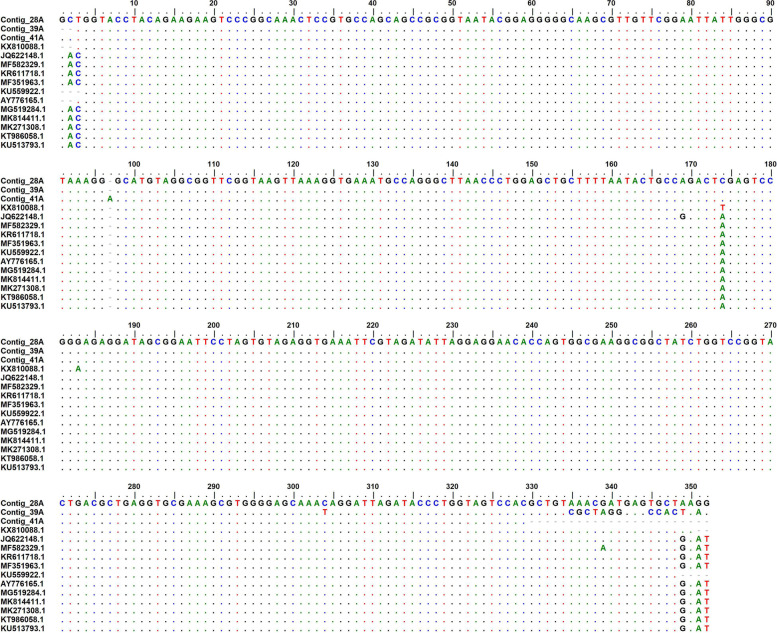
Multiple sequence alignment of 16S rRNA gene for *A. phagocytophilum* isolates. The conserved regions are represented by the dots (.) while the variable regions are indicated by the letters representing the nucleotide A, C, G and T

Nine of the ten PCR products were successfully sequenced on both DNA strands and generated nucleotide sequences with primers EHR16SD/R targeting 345 bp of the 16S rRNA gene of *Anaplasma* species. Multiple alignment of *Anaplasma* nucleotide sequences of three *A. phagocytophilum* isolates revealed that most sequences were conserved expect the following: for our isolates, nucleotides at the first 3 positions and at position 174 differ with the rest of the sequences. At position 304, 39A differs with the rest and at the following positions 378, 381–384,386–388 and 391, 39A and 41A differ with rest (Fig. 2). They all shared 99 to 100% nucleotide similarity (Table 2).

**Table 2:** BLASTn analysis results using 16S rRNA sequences of isolates from olive baboons

Sample number	Animal species	Pathogen identity	E-values	Identity (%)
28A	Olive baboon	A. *phagocytophilum*	4	100
41A	Olive baboon	A. *phagocytophilum*	4	99.39
39A	Olive baboon	A. *phagocytophilum*	3	99.39

**Fig. 3:**
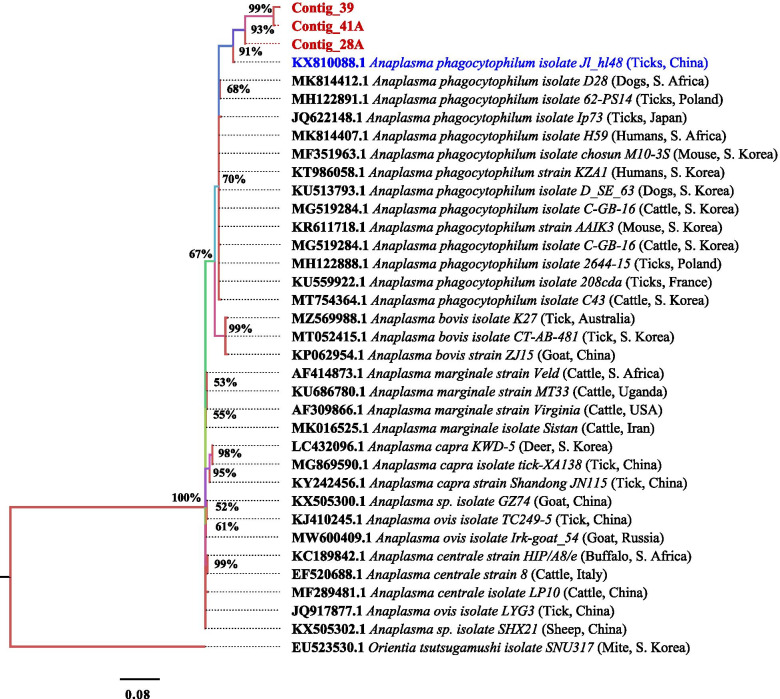
Phylogenetic tree of the 16S rRNA gene from *A.phagocytophilum* isolates

The sequences of the *A. phagocytophilum* isolates from Kenya were identical to those from Japan, South Korea, France, China, South Africa and Denmark. The species isolates were from human, cattle, ticks, dogs and mice. The accession numbers JQ622148, MF351963, KU559922, MG519284, KX810088, MH122888, MK814411, MK814407, MF582329, MK814412, MK271308, MH122891, AY776165, KT986058, KU513793, and KR611718 were all of *A. phagocytophilum* isolates (Fig. 2). Phylogenetic analysis revealed that the isolates from Japan, South Korea, France, China, South Africa, Denmark and Poland belonged to clade I but have recent common ancestor with the Kenyan isolates clustered into one clade II (Fig. 3).

## Discussion

This study is the first to investigate the presence of *A. phagocytophilum* DNA in olive baboons and vervet monkeys in Kenya and it provides molecular evidence of its occurrence in olive baboons within Laikipia County, a wild habitat in close proximity to livestock and humans.

With the rise in reported cases of HGA in United states [24] and exposure of humans to the bacteria [13], detection of this bacteria in primates is a valuable finding since very little information is available in Africa. *Anaplasma phagocytophilum* has been previously reported to infect a wide range of animal hosts in various parts of the world [14–16], including Africa, [17, 18, 25] but from only one study in Kenya [26].

This study found a prevalence of *Anaplasma* spp. of 18.3% (30/164) in olive baboons (17.8%) and vervet monkeys (22.2%) sampled in Laikipia county, Kenya. However, due to limited funding from the project, the current study was limited to sequencing only a few of the gel positive samples. Ten of thirty gel positive samples from olive baboons and vervet monkeys were sequenced which confirmed the presence of *A. phagocytophilum* DNA in three samples from olive baboons in Mpala, Laikipia county, Kenya. This result suggests that if all the 30 samples been sequenced, more *A. phagocytophilum* DNA might have been confirmed even in samples from vervet monkeys and the prevalence of *A. phagocytophilum* could have been determined. Nonetheless, we only report the presence of *A. phagocytophilum* in olive baboons in Kenya.

In this study, primers targeting the 16S rRNA genes of *A. phagocytophilum* were identified from a previous study and used for the detection of *A. phagocytophilum* [21]. The 16S rRNA is a ribosomal RNA gene which are conserved greatly among all bacterial species [27] and are shown to be the most sensitive assay in detection DNA of *A. phagocytophilum* [28]. This makes them very useful in molecular diagnostics. However, 16S rRNA sequences evolve slowly, and while this region is perfect to diagnostic assays, it lacks the population-level variation required when distinguishing genotypes of *A. phagocytophilum* [29, 30]. In this study, the 16S rRNA gene yielded positive bands and therefore reinforced that it is a good marker for detection of *Anaplasma* species as reported by other studies [13, 17, 18]. However, for future studies a longer fragment of 16S rRNA gene should be amplified and obtained with additional primer set for in depth molecular characterization of *A. phagocytophilum.*

Sequencing analysis revealed that the sequences of the 16S rRNA gene were very conserved not only between African isolates but also between the other isolates of world-wide origin agreeably with the only available study on *A. phagocytophilum* in baboons in Zambia [17]. This proved to be a limiting factor to the current study as there is no sufficient data to draw comparison from. The sequences of the *A. phagocytophilum* isolates from Kenya were identical to those from Japan, South Korea, France, South Africa and Denmark and most especially from China (KX810088) in ticks. The species isolates were from cattle, ticks, dogs, rodents and human (Fig. 3). This find was consistent with the study in Zambia [17]. Previous studies have reported genetic relatedness between both *A. phagocytophilum* infecting animals and humans [12, 13].

Multiple alignment of *Anaplasma* nucleotide sequences of the *A. phagocytophilum* isolates revealed that all the sequences of samples from baboons were conserved (Fig. 2). The isolates from Japan, South Korea, France, China, South Africa and Denmark belonged to clade I but have recent common ancestor with the Kenyan isolates clustered into one clade II (Fig. 3). All our isolates were similar to that of ticks from China (KX810088). Amongst them, 39A and 4IA were shown to be evolving much faster than 28A which is because of sequence differences as a result of mutations. Additionally, they are new isolates as suggested by the posterior probability (50%) (Fig. 3).

The presence of *A. phagocytophilum* DNA in baboons in close proximity to humans (wildlife conservancies, ranches) raises the question: to what extent is this bacterial presence in baboon blood a concern for humans? The study localities where the samples were collected is a research center and conservancy with researchers, tourists and local community members who either work or grazed their livestock within. Therefore, this study shows that there is a need for an expansive bio-surveillance of these pathogens with particular attention to these groups of people and their livestock to evaluate the risk of disease transmission in such communities.

Non-human primates have been shown to host different pathogens, including several *Anaplasma* species [17, 31]. We assume that they could serve as a good indicator of bacteria circulation in ecosystem and explain the persistence of anaplasmosis in domestic animals despite consistent control. Therefore, epidemiological surveillance of NHPs’ pathogens is important in generating information that can generate actions developing strategies on prevention and control of emerging and re-emerging zoonotic diseases [17].

## Conclusions

This paper reports the presence of *Anaplasma phagocytophilum* in olive baboons in Laikipia County, Kenya for the first time. This finding open concerns on the public health implications of potentially zoonotic *A. phagocytophilum* infections in the Laikipia community. It proves the need to investigate whether these NHP species are infected with other *Anaplasma* species, raises concerns on the role they play in the maintenance of *Anaplasma* species and therefore, the implications in disease control and prevention.

## Data Availability

Data and materials are available upon reasonable request from the corresponding author.

## Abbreviations

NHPs: Nonhuman primates
USAID: United States AID
PCR: Polymerase chain reaction
DNA: Deoxyribonucleic acid
RNA: Ribonucleic acid
rRNA: Ribosomal Ribonucleic acid
EIDs: Emerging infectious diseases
HGA: Human granulocytic anaplasmosis
DNTPs: Deoxyribonucleotide triphosphate
BLAST: Basic Local Alignment Search Tool
USA: United States of America
